# Oral Prophylaxis

**Published:** 1907-06-15

**Authors:** W. H. Van Deman


### Oral Prophylaxis.

BY W. H. VAN DEMAN, D. D. SC.

My thought in bringiug this subject before you is not to give you anything original, but to try to impress upon you the great need of immediate attention to the subject. I shall not weary you with technique of the treatment, as I hope to show that, as well as the results of the treatment, in my clinic to-morrow. The patient whom I will present came under my care some twenty months ago. At that time the mouth was in as bad condition as one would care to see. The inferior central incisors, as well as the superior second bicuspids and first molars, were floating in pus, the mucous membrane over the entire mouth exceedingly vascular, and the necks of the teeth hyper-sensitive. The general health of the patient was also much impaired. I trust everyone present will take the time to examine this case carefully, and see what beautiful results have been accomplished.

Prophylaxis is not a dream or a barren ideality, but it is one of the most real and vital issues before the profession to-day. It controls the situation more than any other proceeding. It not only rescues the teeth that are well on their way to destruction, but prevents such catastrophies from ensuing. It sets a new and higher standard for every technical operation the dentist makes.

Food can not be masticated properly by teeth that are diseased about their roots, should the patient make ever so determined an effort. The food can not be insalivated by a lot of sore and loose teeth that do not lend themselves readily to the masticating influence. The continual mixing of pyorrhea pus with the food and saliva can not be healthy. When we have once seen a real clean and healthy mouth that has been reclaimed by oral prophylaxis, we can not help but believe in it. It is so different from the usual condition as found in the mouth universally. It seems to me that dentists are asleep in regard to this matter, and I feel that it is my duty to do all that I can to awaken them to the necessity and value of this idea. We must accept and act on this suggestion if we are to keep our place as a branch of the healing art. I have had enough experience with this treatment to be thoroughly convinced of its value and importance, and I want the whole profession to take it up, that we may save the people's teeth and our reputation. It is not an easy work, and any one who does not take it up seriously and do the most thorough work in it, will be disappointed. But by perseverance and tact with skill, the results will abundantly compensate for time and energy spent.

To take on this work means that you will see fewer patients each day, but the results to both patient and operator are so gratifying that better fees can be had; in fact good fees must be had so that the work may be given the first instead of a second place. If every member of this society should go home from this meeting and take up this work, we should accomplish the greatest work in the next year that the profession has ever known. The best way that I know of convincing one of the value of this work is to place yourself in the hands of a competent operator for a prophylaxis treatment. The result will be so convincing that the most skeptical will at once realize the difference between a clean and an unhealthy mouth, and when he takes it up himself and sees the results, he surely will become an enthusiast.

It is necessary that one should equip his office with a suitable outfit of instruments, which need not be expensive, and that he exact fees that will justify the proper amount of time that shall be needed to obtain proper results.

